# Assessment of Dysmyelination with RAFFn MRI: Application to Murine MPS I

**DOI:** 10.1371/journal.pone.0116788

**Published:** 2015-02-13

**Authors:** David Satzer, Christina DiBartolomeo, Michael M. Ritchie, Christine Storino, Timo Liimatainen, Hanne Hakkarainen, Djaudat Idiyatullin, Silvia Mangia, Shalom Michaeli, Ann M. Parr, Walter C. Low

**Affiliations:** 1 Department of Neurosurgery, University of Minnesota, Minneapolis, Minnesota, United States of America; 2 Center of Magnetic Resonance Research, University of Minnesota, Minneapolis, Minnesota, United States of America; 3 A.I. Virtanen Institute for Molecular Sciences, University of Eastern Finland, Kuopio, Finland; Brighton and Sussex Medical School, UNITED KINGDOM

## Abstract

Type I mucopolysaccharidosis (MPS I) is an autosomal recessive lysosomal storage disorder with neurological features. Humans and laboratory animals with MPS I exhibit various white matter abnormalities involving the corpus callosum and other regions. In this study, we first validated a novel MRI technique, entitled Relaxation Along a Fictitious Field in the rotating frame of rank n (RAFFn), as a measure of myelination and dysmyelination in mice. We then examined differences between MPS I mice and heterozygotes using RAFF5 and histology. RAFF5 (i.e., RAFFn with n = 5) relaxation time constants were highly correlated with histological myelin density (R2 = 0.68, P<0.001), and RAFF5 clearly distinguished between the hypomyelinated and dysmyelinated shiverer mouse and the wild-type mouse. Bloch-McConnell theoretical analysis revealed slower exchange correlation times and smaller exchange-induced relaxation rate constants for RAFF4 and RAFF5 compared to RAFF1-3, *T*_1ρ_, and *T*_2ρ_. These data suggest that RAFF5 may assess methylene protons in myelin lipids and proteins, though other mechanisms (e.g. detection of myelin-bound water) may also explain the sensitivity of RAFF5 to myelin. In MPS I mice, mean RAFF5 relaxation time constants were significantly larger for the striatum (P = 0.004) and internal capsule (P = 0.039), and marginally larger for the fornix (P = 0.15). Histological assessment revealed no differences between MPS I mice and heterozygotes in myelin density or corpus callosum thickness. Taken together, these findings support subtle dysmyelination in the brains of mice with MPS I. Dysmyelination may result from myelin lipid abnormalities caused by the absence of α-L-iduronidase. Our findings may help to explain locomotor and cognitive deficits seen in mice with MPS I.

## Introduction

Dysmyelination refers to abnormal myelin structure in the central or peripheral nervous system. The term can be contrast with abnormally low myelin quantity (hypomyelination) and pathological loss of myelin (demyelination). In normal development, oligodendrocyte processes wrap around axon segments to form tight lamellar sheaths. In comparison, myelin is disorganized and irregular in dysmyelination [1,2], leading to deficits in white matter function [3]. Dysmyelinating disorders encompass a wide spectrum of severity and pathophysiology, from leukodystrophies [4] to schizophrenia [5]. Researchers continue to identify anomalous myelin structure in diseases not traditionally associated with white matter [4,6].

Several lines of evidence suggest the presence of myelin pathology in type I mucopolysaccharidosis (MPS I). MPS I is an autosomal recessive lysosomal storage disorder caused by absence of the enzyme α-L-iduronidase (IDUA). Affected children exhibit mental retardation as well as non-neurological features including cardiomyopathy, corneal clouding, and facial abnormalities [7]. Cranial MRI of children with MPS I and other mucopolysaccharidoses reveals variable white matter changes, including cribriform changes in the corpus callosum and other myelinated regions [8–13]. Dogs with MPS I, as well as mice with MPS VII, demonstrate thinning of the corpus callosum relative to unaffected heterozygotes [14,15]. Electron microscopy has revealed abnormal myelin structure in MPS VII [15]. These white matter abnormalities accompany neuronal dysmorphism associated with lysosomal substrate accumulation [16–18].

Mice with MPS I show locomotor and cognitive deficits compared to unaffected heterozygous and wild-type mice. Poor performance on RotaRod testing suggests that MPS I mice have diminished locomotor coordination and sensorimotor integration [19]. Affected mice also show deficits in spatial learning and memory based on Morris water maze [20] and water T maze [21] testing. These particular deficits may be related to variable cerebral distribution of GM3 ganglioside deposits [19].

While sensitive radiological evaluation of myelin in white matter disease is desirable, MRI of myelin is technically challenging. Direct imaging of myelin is difficult because MR signals of myelin lipids and proteins undergo rapid decay and are indistinguishable from those of other tissue elements [22]. A number of strategies have been used to indirectly detect myelin by measuring water trapped in the lamellar structure of the myelin sheath. These methods include standard *T*_1_- and *T*_2_-weighted imaging, MR spectroscopy, diffusion tensor imaging (DTI), magnetization transfer (MT) imaging, and separation of *T*_2_ components [23]. A technique called Relaxation Along a Fictitious Field (RAFF) has recently been introduced as a means for myelin detection [24,25]. This protocol operates in rotating frames of rank n (RAFFn), and has been found to yield high contrast between gray and white matter in ranks 4 (RAFF4) and 5 (RAFF5) [26].

In the present study, we investigated the relationship between myelin content and RAFFn MRI in (1) wild-type mice, (2) severely hypomyelinated, dysmyelinated, myelin basic protein (MBP)-deficient shiverer mice, (3) mice with MPS I, and (4) mice heterozygous for IDUA. We then looked for white matter abnormalities in MPS I mice by comparing RAFF5 relaxation time constants for several gray and white matter regions with unaffected IDUA heterozygotes, which served as controls. Finally, we compared histological myelin density and corpus callosum thickness between MPS I mice and IDUA heterozygotes.

## Materials and Methods

### Ethics statement

All studies were approved by the University of Minnesota Institutional Animal Care and Use Committee (IACUC) under protocols 1405-31519A and 1306-30682A. All efforts were made to minimize animal suffering.

### Animal care and tissue preparation

Mice homozygous for MPS I (IDUA^−/−^) and their heterozygous littermates (IDUA^+/−^) were derived from a colony whose founders were provided by Dr. Elizabeth Neufeld [27,28]. Adult male MPS I mice (N = 6) and IDUA heterozygotes (N = 5) were used in this study. In addition, young (6-week-old) MPS I (N = 1) and IDUA-heterozygous (N = 1) mice were included in a pilot study. Adult female shiverer (C3FeSWV-Mbp-Shi, Charles River, Wilmington, MA; N = 1) and male wild-type (C57BL/6, Jackson Laboratory, Bar Harbor, ME; N = 1) mice comprised the remainder of the study animals. Animals were anesthetized with ketamine (100 mg/kg IP) and xylazine (10 mg/kg IP) and underwent transcardial perfusion with 4% paraformaldehyde. Brains were harvested and cryoprotected in 30% sucrose.

### MRI instrumentation

All MR experiments were performed using a horizontal 9.4 T magnet (Magnex Scientific Ltd., Abington, UK) interfaced to a Varian (Agilent) DirectDrive console (Agilent Technologies, Santa Clara, CA). RF transmission and signal reception were carried out using a quadrature half-volume surface coil with a 20 mm loop diameter (High Field Imaging, Minneapolis, MN). Scout images were collected using *T*_2_-weighted fast spin-echo MR imaging (repetition time *TR* = 4 s, effective echo time *TE* = 55 ms, matrix size 256 × 256, field-of-view = 25.6 × 25.6 mm^2^, 8 echoes with 8 ms echo spacing, initial *TE* = 10 ms, 7 slices with slice thickness 1 mm).

### RAFFn technique

The relaxation in rotating frames 1–5 were measured as described previously [26]. Briefly, RAFFn pulses form four pulse elements that are assembled into a *P*-packet according to the scheme *PP* ^−1^ *P*_π_ *P*_π_^−1^, as utilized previously in adiabatic BIR-4 and RAFFn pulses [24,29]. The time duration of each *PP* ^−1^ *P*_π_ *P*_π_^−1^ packet, defined as $T_{p}=4\pi /(\sqrt{2}\omega_{1}^{\max})$, was set to 2.26 ms to ensure the rotating of **M** to 90° in each of the rotating frames. The signal intensity decay was measured by incremental pulse trains of *P*-packet, with an inversion pulse to account for steady state. The total number of the *PP* ^−1^ *P*_π_ *P*_π_^−1^ packets = 0, 4, 8, 16, 24, 32, leading to pulse train durations from 18 to 145 ms. The peak RF amplitude of RAFFn pulses was set to *γB*_1_ = 625 Hz (RAFF1 and RAFF2), 525 Hz (RAFF3), 323 Hz (RAFF4), or 224 Hz (RAFF5). Readout parameters were identical to those used for scout images.

### Comparative techniques: *T_1_, T_2_, T_1ρ_, T_2ρ_*, and MT

Longitudinal relaxation time constants, *T*_1_ measurements were performed using an inversion recovery technique by adding an inversion pulse and incrementing the inversion time prior to the imaging readout. The hyperbolic secant AFP pulse was applied for inversion, using *T*_p_ = 4 ms, *γB*_1_ = 2.5 kHz, and inversion times of 0.2, 0.5, 0.8, 1.1, 1.4, and 3.0 s. The transverse relaxation time constants, *T*_2_ were measured using an adiabatic double spin-echo preparation block composed of two AFP pulses with the duration *T*_p_ = 3 ms prior to the imaging readout. The echo times in the *T*_2_ measurements were 5, 7, 15, 23, 31, 39, 63 ms.

Adiabatic *T*_1ρ_ and *T*_2ρ_ measurements were performed with adiabatic full passage (AFP) pulses of the hyperbolic secant family, HS1 or HS4 [29] as described previously [30,31]. For the adiabatic *T*_1ρ_ and *T*_2ρ_ measurements, the train of AFP pulses was placed prior to the fast spin echo (FSE) imaging readout. For the *T*_2ρ_ measurements, the AFP pulses were placed after excitation by an adiabatic half-passage (AHP) pulse having a duration *T*_p_ of 4 ms, and a reverse AHP pulse was used to bring magnetization back to the *z*’ quantization axis of the first rotating frame. The pulse trains consisted of a variable number of AFP pulses (0, 4, 8, 24 and 32) with the peak RF amplitude *γB*_1_ = 2.5 kHz and *T*_p_ =3 ms. For the relaxation measurements FSE readout was used with the parameters *TR* = 4 s, effective echo time *TE* = 55 ms, matrix size 256 × 256, field-of-view = 25.6 × 25.6 mm^2^, 8 echoes with 8 ms echo spacing, initial *TE* = 10 ms, 1 slice with slice thickness 1 mm.

For comparison with RAFFn, MT measurements were conducted using modified inversion MT protocol [32]. With MT, the saturation pulse was placed 10 kHz off resonance, and the saturation pulse duration was incremented to obtain longitudinal relaxation time constant *T*_1,sat_ during off resonance saturation, with the pulse durations of 0, 0.3, 0.6, 0.9, and 1.2 s. *γB*_1_ = 200 Hz was used.

### ROI analysis with MRI

For the relaxation mapping with RAFFn, adiabatic *T*_1ρ_ and *T*_2ρ_, MT, and the *T*_1_ and *T*_2_ preparations, fast spin-echo imaging readout was used. The slice of interest was identified from the multi-slice *T*_2_-weighted acquisition. *TR* = 5 s was used. Relaxation time constant maps were calculated in MATLAB (MathWorks, Natick, MA) using the Aedes software package (http://aedes.uef.fi). The following regions of interest (ROIs) were hand-drawn based upon T_2_-weighted images: cortex, lateral septal nucleus, striatum, internal capsule, fornix, and hippocampus (excluding alveus and fimbria). ROIs were copied to the relaxation time constant maps, and mean values were measured for each ROI.

### SWIFT-RAFFn

In addition, the no-echo time SWeep Imaging with Fourier Transform (SWIFT) [33] readout was used for the magnetization prepared with RAFF4 and RAFF5 acquisitions. The SWIFT images were acquired with weighting RAFFn pulse train durations of 9 and 18 ms on *ex-vivo* mouse brains. The RAFFn preparation pulses were inserted every 16^th^ *TR* period as previously described [34] or with same duration of gaps to alter a steady state generated by SWIFT readout. Acquisition parameters were: flip angle 6°, *BW* = 62 kHz, number of projections = 128000, diameter of FOV = 25 mm, and total acquisition time = 12 min, with acquisition 64 complex points during gapped HS2 pulse [35,36] and continuous acquisition of 256 complex points after the pulse. The time delay between the end of acquiring one projection and the start of the next was fixed at 0.6 ms. The field gradients changed values at the beginning of that delay. Each spoke acquisition results in one center-out line of k-space after pre-processing (radial center-out k-space trajectory). 3D radial SWIFT data were processed using an in-house program developed in LabVIEW (National Instruments) and interpolated with a Kaiser-Bessel function onto a Cartesian grid utilizing in-house MATLAB mex code to a matrix of 512^3^ (yielding 0.049 mm nominal resolution).

### Theoretical analysis

Exchange induced relaxations between two spin populations with different chemical shifts (Δ*ω*≠0) during RAFFn and adiabatic *T*_1ρ_ and *T*_2ρ_.were simulated using Bloch-McConnell formalism. The Bloch-McConnell formalism was used as previously described [25]. The power of *T*_1ρ_ was matched with the root mean square of the power used for RAFF2. The rotational correlation time τ_c_ = 10^−12^ s, populations *P*_A_ = 0.9 and *P*_B_ = 0.1, and separation between resonances (Δ*ω*) of 1 ppm at 9.4 T were used for the simulations.

### Histology

Following MRI acquisition, brains of MPS I mice and IDUA heterozygotes were frozen in OCT compound and sectioned horizontally at a thickness of 10 μm. Tissue sections were stained with black gold (Hito, Wilmington, DE), a myelin-specific stain [37]. In the aforementioned pilot study, young mouse brains were sectioned coronally at a thickness of 10 μm and stained with Luxol fast blue (LFB); stained sections from these mice were used for qualitative but not quantitative analysis.

Myelin density was assessed in black gold-stained tissue sections. Mean optical density (OD) was measured in ImageJ (National Institutes of Health, Bethesda, MD) for the following hand-drawn ROIs: slide background, cortex, lateral septal nucleus, striatum, periaqueductal gray, corpus callosum, internal capsule, fornix, subiculum, CA1, CA3, fimbria, and total hippocampus (excluding alveus and fimbria). ROIs were drawn separately in MRI slices and histological sections, based on clearly visible anatomy; no image registration was performed. To account for variable lighting and staining intensity, normalized myelin density was calculated as (OD_ROI_ – OD_background_) / (OD_cortex_ – OD_background_). Normalized myelin density for each ROI was averaged among four tissue sections per mouse to yield values for statistical analysis.

Corpus callosum thickness was also assessed using ImageJ. The anterior-posterior (A-P) midline thickness of the corpus callosum was measured in black gold-stained tissue sections and in the corresponding sections from the 234-section Mikula horizontal brain atlas (http://brainmaps.org, Dataset 9). For each section, the tissue section measurement was divided by the atlas measurement. Normalized A-P midline corpus callosum thickness was averaged among four tissue sections per mouse to yield values for statistical analysis.

### Statistical analysis

All statistical analyses were carried out using GraphPad Prism (GraphPad Software, San Diego, CA). Outliers were defined for each genotype (homozygote and heterozygote) as values more than 1.5 times the interquartile range below the first quartile or above the third quartile. For ROI analysis, data were discarded for mice with outlying values for one third or more of all ROIs. For analysis of corpus callosum thickness, outlying animal means were discarded. Correlation between MRI relaxation time constants and normalized myelin density was assessed with simple linear regression. The two-tailed Student’s t-test was used to assess differences in myelin density and corpus callosum thickness. P values less than 0.05 were considered to be statistically significant.

## Results

### Correlation of MRI with myelin density

**Figure 1:**
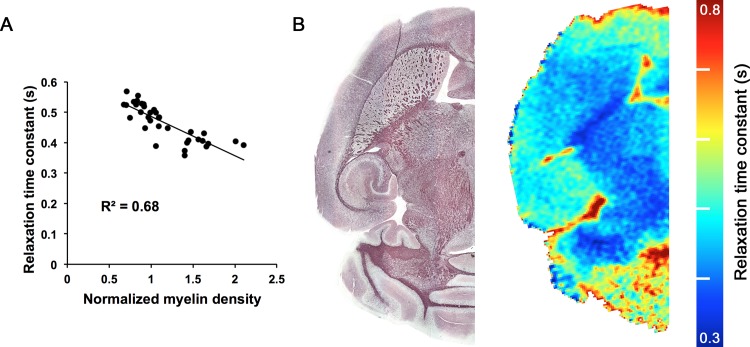
Relationship between RAFF5 and myelin density. **(A)** Correlation of RAFF5 relaxation time constant with normalized myelin density. R^2^ = 0.68. P<0.001. N = 35 (5 ROIs in 7 mice; 4 MPS I mice and 3 IDUA heterozygotes). **(B)** Comparison of black gold histological staining for myelin (left) with RAFF5 relaxation time constant (right), in the horizontal plane, for an IDUA heterozygote.

For each MRI sequence, mean relaxation time constant was compared to normalized myelin density for 5 ROIs (lateral septal nucleus, striatum, hippocampus, internal capsule, and fornix) in 7 mice (4 MPS I mice and 3 IDUA heterozygotes) that underwent both imaging and histological analysis. Normalized myelin density correlated most closely with RAFF5 relaxation time constants (R^2^ = 0.68, P<0.001; Table 1), with an inverse relationship between myelin density and relaxation time (Fig. 1A). Mean relaxation time constant also correlated significantly with normalized myelin density for *T*_1_ (R^2^ = 0.55, P<0.001), *T*_1ρ_-HS1 (R^2^ = 0.37, P<0.001), *T*_1ρ_-HS4 (R^2^ = 0.28, P = 0.001), MT (R^2^ = 0.26, P = 0.002), and RAFF4 (R^2^ = 0.42, P<0.001). Mean relaxation time constant did not correlate significantly with normalized myelin density for *T*_2_ (R^2^ = 0.07, P = 0.13), *T*_2ρ_-HS1 (R^2^ = 0.10, P = 0.07), or *T*_2ρ_-HS4 (R^2^ = 0.02, P = 0.43).

**Table 1:** Correlation of MRI relaxation time constants with normalized myelin density.

**MRI sequence**	**R^2^**	**P**
*T*_1_	0.55	<0.001
*T*_1ρ_-HS1	0.37	<0.001
*T*_1ρ_-HS4	0.28	0.001
*T*_2_	0.07	0.13
*T*_2ρ_-HS1	0.10	0.07
*T*_2ρ_-HS4	0.02	0.43
MT	0.26	0.002
RAFF4	0.42	<0.001
RAFF5	0.68	<0.001

The similarity between the RAFF5 relaxation time constant map and histological myelin staining is demonstrated in Fig. 1B. Because mean RAFF5 relaxation time constant correlated most closely with normalized myelin density, we elected to use RAFF5 to evaluate for myelin differences between MPS I mice and IDUA heterozygotes.

### RAFF5 in a known hypo/dysmyelination model

**Figure 2:**
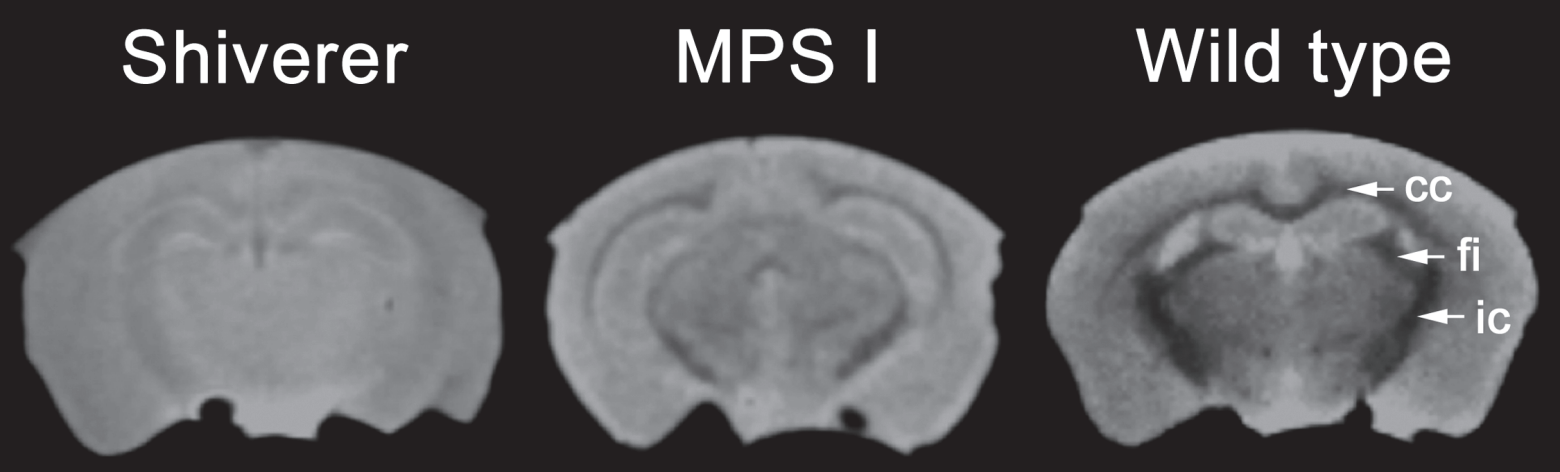
RAFF5 comparison of a congenitally hypo/dysmyelinated shiverer mouse, an MPS I mouse, and a wild-type mouse. Coronal images were obtained with RAFF5 weighting to SWIFT imaging readout. cc, corpus callosum; fi, fimbria; ic, internal capsule.

Fig. 2 shows representative images, obtained with RAFF5 preparation pulses using SWIFT imaging readout, from (1) the congenitally hypomyelinated and dysmyelinated shiverer mouse, (2) a mouse with MPS I, and (3) a wild-type control. Contrast between gray and white matter regions is essentially nonexistent in the shiverer mouse. In comparison, myelinated white matter tracts such as the corpus callosum, fimbria, and internal capsule are clearly distinguishable in the wild-type mouse. These tracts are visible in the MPS I mouse as well, but contrast between gray and white matter is diminished relative to the wild-type mouse.

### Theoretical analysis using Bloch-McConnell equations

**Figure 3:**
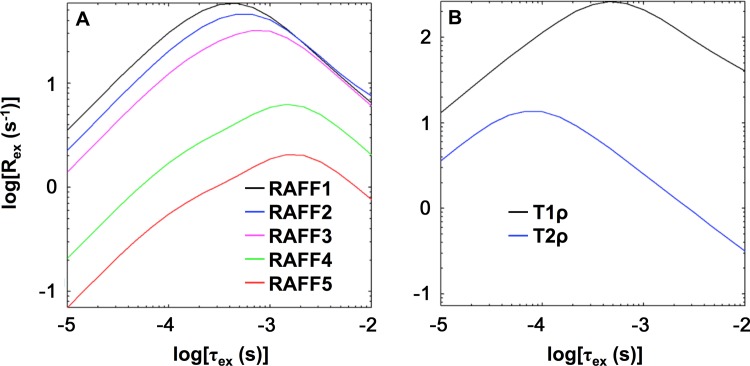
Bloch-McConnell theoretical analysis. Plots depict exchange-induced relaxation rate constant (R_ex_) versus exchange correlation time (τ_ex_) for **(A)** RAFFn and **(B)** adiabatic *T*_1ρ_ and *T*_2ρ_. Bloch-McConnell equations were used as previously described [25]. Refer to text for parameter values.

Simulated exchange-induced relaxation rate constants are shown in Fig. 3. The maximal exchange-induced relaxation rate constants (R_ex_) of RAFFn shifted towards slower exchange correlation times (τ_ex_) with increasing n (Fig. 3A). The value of τ_ex_ corresponding to maximal R_ex_ was 0.4 ms for RAFF1, 0.5 ms for RAFF2, 0.8 ms for RAFF3, 1.1 ms for RAFF4, and 1.1 ms for RAFF5. Adiabatic *T*_1ρ_ (peak R_ex_ at τ_ex_ = 0.4 ms) had a sensitivity range similar to RAFF1-3, but the relaxation rate constants were much larger (Fig. 3B). The sensitivity range of adiabatic *T*_2ρ_ extended to faster τ_ex_ (peak R_ex_ at τ_ex_ = 0.08 ms) than adiabatic *T*_1ρ_.

### RAFF5 relaxation time constant in MPS I

**Figure 4:**
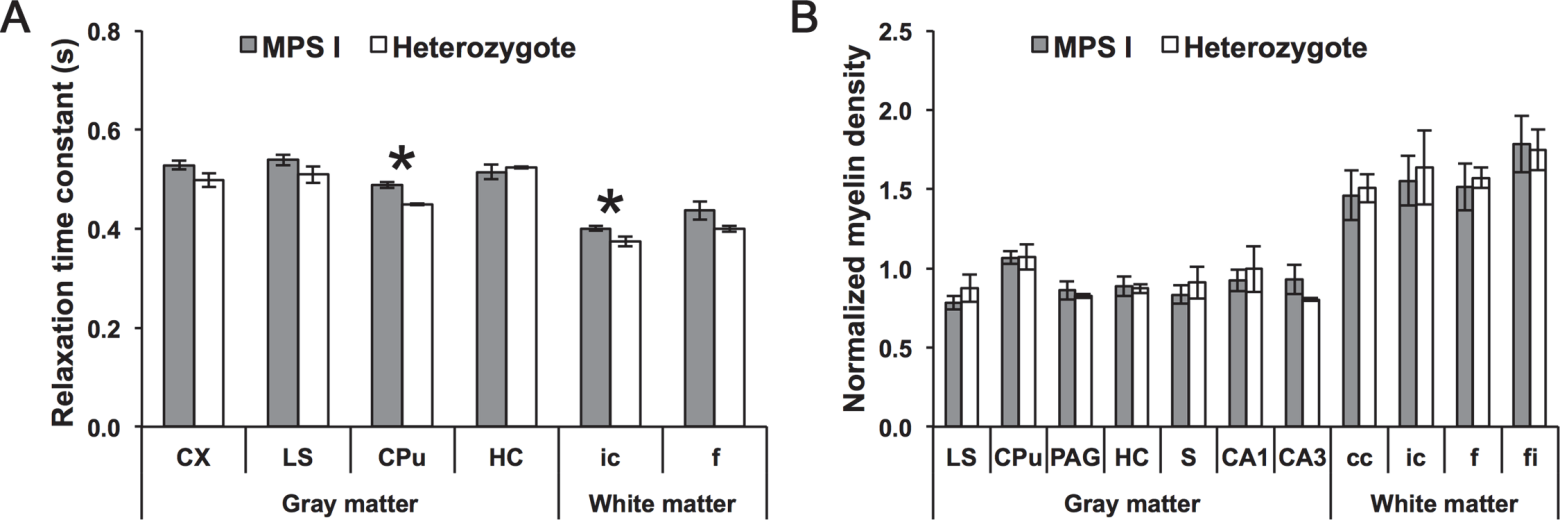
RAFF5 and myelin density, by ROI, in MPS I mice and IDUA heterozygotes. **(A)** RAFF5 relaxation time constant. N = 7 mice (4 MPS I mice and 3 IDUA heterozygotes). **(B)** Normalized myelin density. N = 8 mice (5 MPS I mice and 3 IDUA heterozygotes). *P<0.05. Error bars represent ±1 standard error. cc, corpus callosum; CPu, striatum; CX, cerebral cortex; f, fornix; fi, fimbria; HC, hippocampus; ic, internal capsule; LS, lateral septal nucleus; PAG, periaqueductal gray; S, subiculum.

RAFF5 relaxation time constant means for 6 ROIs was compared between 5 MPS I mice and 4 IDUA heterozygotes (Fig. 4A). Of these mice, 2 mice (1 MPS I mouse and 1 IDUA heterozygote) were excluded from this comparison due to outliers (refer to Materials and Methods for exclusion criteria). Mean relaxation time constants were larger in MPS I mice than in IDUA heterozygotes for the striatum (P = 0.004) and internal capsule (P = 0.039), and did not significantly differ by genotype for the cerebral cortex (P = 0.11), lateral septal nucleus (P = 0.17), hippocampus (P = 0.64), or fornix (P = 0.15).

### Myelin density in MPS I

Normalized myelin density for 11 ROIs were compared between 5 MPS I mice and 4 IDUA heterozygotes (Fig. 4B). Of these mice, 1 IDUA heterozygote was excluded from this comparison due to outliers. Myelin density did not vary for any regions, including the lateral septal nucleus (P = 0.32), striatum (P = 0.95), periaqueductal gray (P = 0.66), hippocampus (P = 0.91), subiculum (P = 0.49), CA1 (P = 0.64), CA3 (P = 0.34), corpus callosum (P = 0.85), internal capsule (P = 0.77), fornix (P = 0.78), or fimbria (P = 0.90).

### Corpus callosum thickness in MPS I

**Figure 5:**
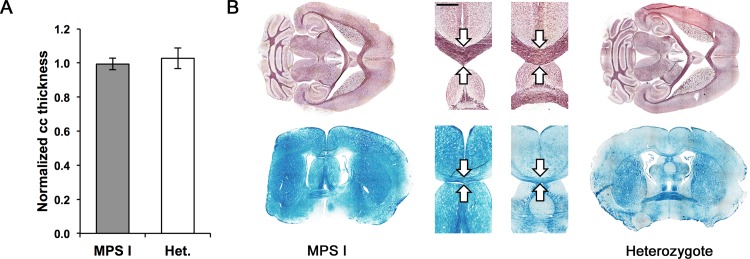
Corpus callosum thickness in MPS I mice and IDUA heterozygotes. **(A)** Anterior-posterior thickness of corpus callosum (cc) on midline, relative to atlas, in MPS I and IDUA heterozygote mice. P = 0.65. N = 8 mice (4 MPS I mice and 4 IDUA heterozygotes). Error bars represent ±1 standard error. **(B)** Corpus callosum thickness in MPS I mice (left) and IDUA heterozygotes (right). Top: horizontal, black gold-stained sections from adult mice. Bottom: coronal, LFB-stained sections from young mice. Inset: magnification of corpus callosum (arrows) showing no difference in thickness. Scale bar represents 1 mm for inset.

Anterior-posterior midline thickness of the corpus callosum was measured in 5 MPS I mice and 4 IDUA heterozygotes and normalized to atlas measurements (Fig. 5A). Of these mice, 1 MPS I mouse was excluded from this comparison due to its outlying value. Normalized corpus callosum thickness did not vary between MPS I mice and IDUA heterozygotes (P = 0.65). Fig. 5B shows the similar appearance of the corpus callosum in MPS I mice and IDUA heterozygotes in horizontal and coronal planes.

## Discussion

In the present study, we validated the novel MRI technique called RAFFn as a measure of myelination and applied this technique to assess for myelin abnormalities in mice with MPS I. RAFF5 was closely correlated with myelin density and easily distinguished between normal and severely dysmyelinated brains. Compared to IDUA heterozygotes, mice with MPS I exhibited larger RAFF5 relaxation time constants for the striatum and internal capsule. However, histological analysis revealed no differences in myelin density or corpus callosum thickness. These results indicate the presence of dysmyelination in murine MPS I and may in part account for neurological deficits previously observed in these mice.

### Myelin assessment with RAFFn MRI

Previous research has shown that RAFFn yields high contrast between gray and white matter in high-rank rotating frames (RAFF4 and RAFF5) [26]. We found high correlations with histological myelin density for RAFF4 (R^2^ = 0.42) and RAFF5 (R^2^ = 0.68) relaxation time constants. Interestingly, only conventional T_1_-weighted MRI (R^2^ = 0.55) showed a similarly close relationship with myelin content. RAFF5 also revealed near-total loss of contrast between gray and white matter in the shiverer mouse. Shiverer is an MBP mutation characterized by severe hypomyelination [38] and non-compact myelin sheaths [2] with preservation of normal axonal tracts [39]. The clear difference between RAFF5 maps for wild-type and shiverer mice implies that RAFF5 is capable of detecting hypomyelination, dysmyelination, or both.

The basis of myelin detection by high-rank RAFFn MRI is not entirely clear. Direct detection of myelin lipids and proteins is complicated by extremely rapid signal relaxation as well as poor differentiation from other tissue types [23]. In myelin, three populations give rise to the *T*_2_ signal: methylene protons (*T*_2_ from 50 μs to 1 ms), water trapped between myelin lipid layers (*T*_2_≈20 ms), and free intracellular and extracellular water (*T*_2_≈100 ms) [23,40]. Several techniques have been developed to assess myelin by detecting motion-restricted water in the lamellar myelin structure [23].

Bloch-McConnell simulations of RAFFn, *T*_1ρ_, and *T*_2ρ_ revealed trends that may help to explain variation in myelin sensitivity. Exchange-induced relaxation rate constants of RAFFn shifted towards slower exchange correlation times with the increasing n; this finding was expected because lower power is used for RAFFn pulses in high frames. RAFF4 and RAFF5 exhibited slower exchange correlation times and smaller exchange-induced relaxation rate constants compared to RAFF1-3 as well as *T*_1ρ_ and *T*_2ρ_. We have previously shown that the decrease of exchange-induced relaxation rate constants in high frames is larger for slow exchange rather than for fast exchange. In addition, the tip angle of **M** and the specific absorption rate decrease during RAFFn pulses with the increase of n [26]. These features of RAFFn at high rotating frames may capture fast-relaxing spins—including methylene protons—that escape detection with other methods.

Other relaxation pathways may contribute to the myelin sensitivity of RAFFn. Of note, RAFF4 and RAFF5 correlate with myelin density similar to *T*_1_ and much more than MT. RAFFn and *T*_1_ may share isochronous mechanisms (i.e., exchange between spins with identical chemical shifts) and dipolar relaxation pathways and cross-relaxations. While important, the consideration of these relaxation pathways is outside of the scope of current work.

### Evidence for dysmyelination in MPS I

After validating RAFF5 for assessment of myelination and dysmyelination, we compared mean RAFF5 relaxation time constants for several gray and white matter regions between affected, homozygous MPS I mice and unaffected IDUA heterozygotes. In MPS I mice, relaxation time constants were longer for the striatum and internal capsule, but did not significantly differ for other regions (cerebral cortex, lateral septal nucleus, hippocampus, and fornix). Histological staining for myelin demonstrated no differences in the quantity and gross structure of myelin, excluding the possibility of hypomyelination in MPS I mice.

We concluded above that larger mean relaxation time constants reflect either decreased myelin quantity (hypomyelination) or quality (dysmyelination). Given that myelin density did not differ between MPS I mice and IDUA heterozygotes, we reason that the differences observed using RAFF5 MRI reflect subtle dysmyelination in the major white matter tracts of MPS I mice. If RAFF5 detects myelin lipids and proteins, increased relaxation time constants may result from abnormal macromolecular composition in the myelin of MPS I mice. Alternatively, if RAFF5 reflects myelin-bound water, less water motion restriction—due to loss of the compact myelin structure in dysmyelination—could increase relaxation time constants. One or both of these mechanisms may explain why dysmyelinated regions have larger mean RAFF5 relaxation time constants that are more characteristic of gray matter than white matter.

White matter features of MPS I seem to vary from species to species. The corpus callosum in MPS I-affected humans exhibits primarily cribriform defects [8,9], whereas dogs with MPS I demonstrate marked thinning of the corpus callosum without cribriform changes [14]. Mice with MPS VII likewise show substantially reduced corpus callosum thickness [15], but no published study to date has reported corpus callosum abnormalities in MPS I. We observed no gross corpus callosum abnormalities in MPS I mice, and the thickness of the corpus callosum did not differ from unaffected IDUA heterozygotes. This discrepancy suggests that future investigation of RAFFn MRI in humans with MPS I is warranted.

### Mechanism and significance of dysmyelination in MPS I

The enzyme IDUA catalyzes breakdown of glycosaminoglycans (GAGs) containing α-L-iduronic acid [41]. Absence of IDUA in MPS I leads to accumulation of various gangliosides in brain and other tissues [19,42]. Of these gangliosides, GD3 and GM1 are enriched in oligodendrocytes and myelin membranes [43,44]. Buildup of these molecules in myelin may produce dysmyelination in MPS I. This possibility is supported by its similarity to myelin pathology in NCTR-Balb/C mice. These mice possess a different lysosomal storage disorder in which accumulation of sphingomyelin and other myelin lipids produces a dysmyelinating phenotype [45,46]. The distribution of GM3 ganglioside provides further support for a relationship between ganglioside deposits and dysmyelination. In MPS I mice, the striatum—which we found to be the most significantly dysmyelinated brain region—contains the highest abundance of GM3 [19].

Previous studies have revealed that MPS I mice possess locomotor and cognitive deficits not seen in heterozygous littermates or wild-type mice. Mice with MPS I perform poorly on the RotaRod test and do not improve over time, indicating abnormalities of both coordination and motor learning [19]. MPS I mice also fare worse in the Morris water maze and water T maze tests, reflecting spatial learning and memory deficits [20,21]. The regions of putative dysmyelination that we have identified are critical for locomotion and cognition. The striatum and internal capsule play central roles in movement and coordination. The fornix—which differed marginally (P = 0.15) on RAFF5 MRI between MPS I mice and IDUA heterozygotes—is a key element of the Papez circuit that is responsible for memory formation [47]. The hippocampus, which is also essential for learning, did not vary between affected and unaffected mice on MRI; however, this was to be expected given that the hippocampus is rich in unmyelinated axons [48].

Behavioral deficits in murine MPS I have previously been attributed to neuronal pathology, and nonmyelinated motor (cerebral and cerebellar cortex) and limbic (hippocampus) areas demonstrate highest IDUA expression following gene therapy for MPS I [20]. However, the findings of the present study suggest that subtle dysmyelination may also contribute to neurological features of MPS I.

### Future work

Subsequent laboratory and clinical studies can help to clarify the significance of our results. Electron microscopy of white matter areas is needed to provide ultrastructural proof of dysmyelination. Biochemical assessment of the relationship between myelin ganglioside content and dysmyelination may provide insight into myelin pathophysiology in MPS I. RAFF5 MRI of patients with MPS I can establish the translation of our findings to the human disease. Finally, RAFFn may be used to investigate myelination and myelin pathology in other conditions.
